# Factors Affecting Flock Uniformity in Broiler Production: Individual, Environmental, and Management Characteristics

**DOI:** 10.3390/ani16020185

**Published:** 2026-01-08

**Authors:** Janghan Choi, Doyun Goo, Hanseo Ko, Jihwan Lee, Woo Kyun Kim

**Affiliations:** 1 Department of Animal and Food Sciences, Davis College of Ag. Sciences & Natural Res., Texas Tech University, 1308 Indiana Ave., Lubbock, TX 79409, USA; 2 Department of Poultry Science, University of Georgia, Athens, GA 30602, USA; dgoo@uga.edu (D.G.); hsko@uga.edu (H.K.); wkkim@uga.edu (W.K.K.); 3 Department of Animal Sciences, Jeonbuk National University, Jeonju 54896, Republic of Korea; jl26112@jbnu.ac.kr

**Keywords:** broilers, flock uniformity, gut health, environmental effects, microbial infection, nutrition

## Abstract

Flock uniformity is a critical factor influencing productivity, production efficiency, animal welfare, and overall economic outcomes in broiler production. Despite its importance, uniformity is often overlooked in commercial settings, where production goals typically focus on maximizing average BW and overall flock performance rather than minimizing variation among individual birds. This review aims to summarize the diverse factors that affect flock uniformity in broilers. Under standard conditions between days 14 and 49, flock uniformity typically reaches approximately 90%. Both intrinsic factors, such as genetics, initial BW, age, sex, and rearing method, and extrinsic factors, including housing, management, and vaccination practices, have been shown to influence uniformity. Environmental and management challenges, such as heat stress, nutrient deficiencies, microbial infections, high stocking density, feed form, and mixed-sex rearing, can further reduce uniformity by 2.5–15%. These findings highlight the high sensitivity of flock uniformity to multiple stressors, which may interact rather than act independently. Moreover, these findings highlight the need to consider both baseline and challenging factors in broiler production and support the development of targeted management and environmental strategies to improve flock uniformity and enhance overall production efficiency.

## Simple Summary

Flock uniformity refers to how closely birds within the same group match in body weight (BW). This characteristic is important because it influences production efficiency, processing accuracy, animal welfare, and overall economic returns within modern broiler operations. Despite its importance, flock uniformity is often overlooked in commercial systems because management decisions commonly focus on increasing average BW rather than limiting variation among individual birds. This review explains the main factors that affect how similar broiler chickens are in BW, a trait known as flock uniformity. Under typical commercial conditions between D 14 and 49, most flocks reach about 90% uniformity. Differences in genetics, initial weight, age, sex, rearing method, housing, management, and vaccination all play a role in creating variation among birds. Environmental and management challenges such as heat, nutrient shortages, infections, crowded pens, feed type, and mixed-sex flocks can reduce uniformity, showing that flocks are sensitive to multiple factors working together. Reduced uniformity creates challenges for feeding programs, increases the risk of welfare concerns for smaller birds, and decreases the efficiency of automated processing systems. By understanding how these diverse factors influence flock uniformity, producers and researchers can develop management approaches that support more consistent performance, improve animal welfare, and enhance productivity across the poultry industry.

## 1. Introduction

Intense genetic selection, combined with an effective feeding program and efficient rearing management greatly improved productivity and efficiency of broiler production [1]. Despite the low genetic variation and well-controlled rearing environment in modern broiler production, there can still be noticeable variations in body weight (BW) within a flock [2]. Ideally, broiler production wants to produce homogenous products for production efficiency [3]. High variations in BW within a flock (e.g., poor uniformity) can cause tremendous economic loss mainly by decreasing efficiency feeding programs, increasing mortality, and inducing slaughterhouse rejection [4,5]. This is because, nutrient requirements and nutritional programs are built based on specific BW and age of broiler chickens [6]. The automated processing line in the slaughterhouse endeavors to process broilers with similar BW, and a surplus of broilers outside the designated range would induce economic loss [7]. In addition, poor uniformity can raise animal welfare concerns, as smaller or weaker birds may experience chronic stress, competition for feed and water, uneven access to resources, and higher susceptibility to disease or injuries [5].

Flock uniformity is commonly quantified using the coefficient of variation (CV) of BW, calculated as: Coefficient of variation (CV) (%) = $\frac{SD\;of\;BW}{Mean\;of\;BW}$ × 100, where SD of BW is the standard deviation (SD) of individual BW in the flock, and Mean BW is the average BW in the flock. A lower CV indicates a more uniform flock. Flock uniformity rate is calculated using the formula: 100%−CV(%). Flock uniformity is often further expressed as the percentage of birds within ±10% of the mean BW, which is a key indicator for product consistency [5]. In modern broiler flocks, CV of BW typically ranges from 10% to 15% at market age, highlighting the persistent challenge of maintaining uniformity even under controlled conditions [8].

Diverse factors including genetics, rearing environments (e.g., temperature and humidity), water and feed accessibility, and feed composition can affect flock uniformity in broilers [4,9]. While uniformity is a universal challenge in poultry production, uniformity is often neglected in commercial settings because production goals typically prioritize maximizing average BW and flock performance rather than minimizing variation among individual birds. Moreover, cost-effective interventions to improve uniformity remain limited with culling of extremely light birds being the most commonly applied approach [5]. There is a pressing need for effective strategies to enhance flock uniformity. Achieving this requires a comprehensive understanding of the factors influencing flock uniformity including those present under normal conditions as well as those that arise under challenging situations. Therefore, the purpose of the review is to systematically examine the diverse factors affecting flock uniformity in broilers and discuss how these factors interact to shape flock uniformity.

## 2. Factors Influencing Flock Uniformity

### 2.1. Genetics and Initial BW

Over the past 60 years of narrow and intense genetic selection, modern broilers are known to have less genetic variations [3,10]. However, several studies reported that there were genetic variations in residual variations (e.g., unexplained variations) in livestock animals [3]. A previous study by Rowe et al. [11] showed that there was significant genetic heterogeneity of residual variance in BW among roosters, which can potentially contribute to variations in egg size and BW among their offspring. In livestock animals, although the heritability of residual variance is generally low (0.02 to 0.05), the ratio of genetic standard deviation to the population average residual variance can reach up to approximately 0.5. Genetic heterogeneity in residual variance contributes to variations in BW among individuals, which can affect uniformity at the flock level. Therefore, genetic factors could be important contributors to BW variation within a flock [12,13]. In addition, flock uniformity may differ among strains, as previous studies have shown that different strains can exhibit distinct growth patterns and physiological characteristics [13,14]. A previous study by Pascalau et al. [15] showed that Ross and Cobb strains exhibited potential differences in flock uniformity. Therefore, genetics can be considered one of the key factors affecting flock uniformity in broiler production.

Along with genetics, egg size and initial BW could be a congenital factor that can influence flock uniformity in broiler production. According to our internal data (unpublished), the BW of day-old chicks ranges from approximately 25 g to 50 g. Numerous factors within the broiler breeder system can affect egg size uniformity, and egg weight is highly correlated with the initial BW of chicks [16,17]. Nonetheless, Pinchasov [18] demonstrated that the effects of large eggs on chicks’ BW disappeared rapidly after hatching with feed intake emerging as the major influencing factor on BW. Moreover, Neto et al. [19] demonstrated that grouping broiler chicks by initial BW, as opposed to random allocation, did not result in increased uniformity of broilers at the final BW. Egg size directly influences initial BW of chicks, but its effect on final BW may diminish due to post-hatch growth factors. Hence, although initial BW may affect early growth, it does not necessarily lead to improved flock uniformity at growing and finishing phase. Therefore, while egg size and initial BW influence early growth, management and genetic factors play a more critical role in determining flock uniformity at later stages.

### 2.2. Age

Broilers exhibit rapid growth within a relatively short production cycle of approximately 42–56 days [20]. Depending on market objectives (e.g., whole-bird marketing or cut-up parts) and strategic considerations such as disease control or the reduction of breast muscle myopathies, the slaughter age of broilers worldwide typically ranges from 28 to 56 days [21,22]. Several studies demonstrated that flock uniformity is significantly and positively correlated with increasing age in broilers, suggesting that birds tend to become more homogeneous in BW as they progress through the production cycle [19,23,24]. Younger chickens are more prone to BW variations due to their heightened susceptibility to environmental and pathogenic challenges, as well as immature immunological capacity [25], which can amplify initial disparities in BW. Moreover, early growth phases are characterized by high relative growth rates, causing minor differences in feed intake or pathogen susceptibility to rapidly diverge and contribute substantially to flock uniformity. As broilers age, their capacity for feed intake increases, allowing slower-growing individuals to partially compensate for and improve flock uniformity over time. Furthermore, maturation of the immune system and improvements in gut stability lessen susceptibility to enteric disturbances [26]. Nevertheless, it is worth noting that heat stress can still impose considerable variability during the grower–finisher phase by reducing feed intake, impairing thermoregulation, and limiting growth potential, ultimately contributing to decreased flock uniformity in older birds under high-temperature conditions [27]. Moreover, contrasting evidence exists, as other studies have reported a decline in flock uniformity with advancing age, indicating that variability in growth rates may become more pronounced over time [28,29,30]. These contradictory findings highlight that age-related changes in uniformity would be influenced by multiple interacting factors, such as management practices, genetics, health status, and environmental conditions. Taken together, because flock uniformity is influenced by age-dependent physiological and environmental factors, uniformity should be considered as an important criterion when determining slaughter age under various production conditions.

### 2.3. Same Sex or Mixed Sex Rearing

Although male and female broilers differ in growth performance, nutrient requirements, and physiology, mixed-sex (straight-run) rearing has become more common in commercial production because sexing is time-consuming and requires skilled labor [9]. Most studies comparing single-sex and mixed-sex rearing were conducted more than 50 years ago using less intensively selected breeds, and their findings on growth performance and uniformity were inconsistent [31]; however, recent studies have provided updated evidence, which are summarized in Table 1.

The results from the summarized studies suggests that (1) the only male rearing had the highest final BW and followed by straight-run rearing and lastly the only female rearing; and (2) the only female rearing had the highest uniformity and followed by the only male rearing and lastly the straight-run rearing [33,35]. The lower final BW and improved uniformity observed in female-only flocks may be attributed to reduced competition for feed intake compared to mixed-sex flocks, where male broilers typically dominate access to feed. Furthermore, the reduced uniformity in the straight-run rearing would be potentially because of the higher feed intake desire of male broilers, which could lead to their dominance over female broilers [34]. England et al. [34] showed that straight-run rearing decreased flock uniformity in female chickens. This would be because nutrient requirements being established primarily for male chickens, resulting in relative nutrient deficiencies for females and consequently lower uniformity. Whereas there were not many recent studies on the uniformity with single-sex or straight run rearing, it still has been shown that there were discrepancies in the results of uniformity and growth performance depending on single-sex or straight-run rearing. Additional research is needed (1) to ascertain the impacts of single-sex or straight-run rearing on flock uniformity and productivity within modern broiler production systems; and (2) to find solutions to improve flock uniformity in straight-run rearing.

### 2.4. Housing and On-Farm Management

Factors related to housing and on-farm management including bedding type, air quality, feeding management, and lighting, may influence overall flock performance and uniformity. Vasdal et al. [5] demonstrated that flock uniformity was significantly correlated with growth rate and mortality, suggesting that practical on-farm management plays a critical role in determining flock uniformity. However, litter score and bedding type were not directly correlated with flock uniformity, indicating that not all environmental factors exert the same influence.

Within lighting management, light uniformity is considered a key factor in broiler production, together with light intensity and light source characteristics [36,37,38]. Light plays a crucial role in regulating feeding behavior, general management, and stress responses in broilers [39]. Variations in light distribution within the house may therefore contribute to differences in flock uniformity. Galosi et al. [29] reported that the use of LED lighting improved flock uniformity compared with conventional lighting systems. A previous study by Griffin et al. [40] demonstrated that longer rearing light period decreased growth rate and flock uniformity of broilers. Therefore, uneven light distribution and inappropriate lighting programs can contribute to variability in growth and behavior among broilers, ultimately affecting flock uniformity.

Feeder type and feeding management are well known to affect flock uniformity in broiler breeders, primarily through feed restriction strategies [41]. In contrast, broilers are typically fed ad libitum, and feeder type alone may not markedly influence flock uniformity. However, feeder height, feeder accessibility, and feeder distribution within the house may still influence feeding behavior and contribute to BW variation among birds. In addition to feeding management, water availability and water quality are critical components of on-farm management that may interact with feeding behavior and nutrient intake [42]. Limited access to drinkers or uneven drinker distribution may reduce water consumption in certain birds, indirectly affecting feed intake and growth performance. Moreover, poor water quality or contamination of feeders and drinkers may elicit variable individual responses due to differences in health status and physiological sensitivity among broilers [43]. Such variability in feed and water intake patterns may ultimately exacerbate differences in nutrient utilization and contribute to reduced flock uniformity.

Air quality, particularly ammonia concentration, is known to affect broiler performance and health [44]. Variations in ventilation efficiency and microclimatic conditions within a house may result in uneven exposure among birds, potentially leading to differences in flock uniformity [45]. In addition, individual birds may respond differently to ammonia exposure due to differences in physiological sensitivity, health status, and adaptive capacity [46]. Beyond the factors described above, variations in other environmental conditions such as within the house may also influence flock uniformity. Importantly, birds do not respond uniformly to these conditions, and individual differences in sensitivity may further exacerbate variation in growth and performance [47]. These effects may be particularly pronounced during the early brooding and growth phases, when young birds are more sensitive to environmental fluctuations [48]. Overall, housing conditions and on-farm management practices can influence flock uniformity; however, further research is required to clarify the relative contribution and interaction of individual management factors and individual bird responses across different production stages.

### 2.5. Stocking Density

Definition of stocking density is the number of chickens or total live weight in a fixed area, and stocking density is closely associated with the production efficiency and animal welfare [49]. Many producers have tried to adopt the highest stocking density in modern broiler production to yield the highest profits per fixed area [50]. It is well-known that excessively high stocking density can lead to decreased growth performance, reduced feed intake, increased inflammation and oxidative stress, and increased susceptibility to heat stress in broiler production [51,52]. It can be hypothesized that high stocking density can increase BW variance because dominant individuals may continuously occupy feed access compared to the small size of broilers. However, a previous study by Goo et al. [51] showed that increasing stocking density (15.2 birds/m^2^ to 30.4 birds/m^2^) did not affect flock uniformity on D 28. Moreover, Franco-Rosselló et al. [53] reported that flock uniformity was not affected by stocking density (27 kg BW/m^2^ to 39 kg BW/m^2^) on D 42. At the large-scale studies in many practical farms, flock uniformity was not correlated with the stocking density D 42 [5]. Similarly, Kwon et al. [54] reported that stocking densities of 16.7 and 20.2 birds/m^2^ in large-scale farms (30,000 birds per house) did not influence flock uniformity. Nevertheless, Feddes et al. [8] demonstrated that lower stocking densities (11.9 to 23.8 birds/m^2^) can decrease flock uniformity during the finisher phase (days 38 and 42), as the additional space allows fast-growing broilers to reach their maximum growth potential and outcompete smaller birds. Despite ongoing debate, flock uniformity tended to be lower in younger and smaller broilers than in older and larger ones, implying that lower stocking density (approximately 12–16 birds/m^2^ according to previous studies) could negatively influence flock uniformity [53]. In summary, while high stocking density does not consistently impair flock uniformity in broilers, low stocking density can lead to decreased uniformity.

### 2.6. Nutrient Deficiency and Feed Form

The nutrient composition and physical form of feed can significantly influence the growth performance of broiler chickens [55]. Reducing crude protein (CP) levels in broiler diets with supplementation of limiting amino acids, has become a common strategy to reduce feed costs and improve nitrogen utilization in broiler production [56]. Reducing dietary energy levels is generally avoided because of its negative impact on growth performance and feed efficiency [57,58], although some studies have reported no adverse effects under certain conditions [59,60]. A previous study by Saleh et al. [61] demonstrated that decreasing CP in broiler diets may not affect growth performance and flock uniformity of broiler chickens. However, low CP diets can negatively affect growth performance and flock uniformity of broiler chickens depending on the experimental conditions and reduction levels. While Ojediran et al. [62] reported that low CP (25% of reduction) level did not affect final BW and flock uniformity in broilers, Ahiwe et al. [63] showed that the low energy (3% of reduction) and protein levels (3% of reduction) in the feed reduced flock uniformity with reduced nutrient digestibility on D 21 and D 42. Moreover, Corzo et al. [64] demonstrated that the low amino acid concentration in the feed reduced flock uniformity. A large-scale study demonstrated that flock uniformity was negatively correlated with poor feed efficiency and positively correlated with growth rate in broiler chickens on D 42 [5], indicating that differences in growth caused by nutrient deficiencies can be a primary factor affecting flock uniformity. In addition, Behre and Gous [65] showed that flock uniformity can be exacerbated not only by poor nutrient utilization but also by individual differences in the willingness to consume more feed when faced with nutrient deficiencies. Nonetheless, a previous study by Rubio et al. [23] demonstrated that feed mixing time and feed uniformity did not significantly affect flock uniformity. While feed uniformity is an important factor, if the feed is adequately mixed, it has little impact. Hence, nutrient deficiencies can reduce flock uniformity both by limiting nutrient utilization and by inducing individual differences in feed intake and growth responses.

Modern broilers are normally fed with pelleted or crumbled feeds rather than mash feed because pelleted or crumbled feeds are known to improve BW, feed intake, and feed efficiency [60,66]. A previous study by Xu et al. [67] involving pelleted or crumbled feed improved broiler flock uniformity on D 14, and the potential mode of actions would be (1) increasing feed intake; (2) enhancing nutrient utilization; (3) improving feed homogeneity; and (4) decreasing selective feeding behavior. A previous study by Xu et al. [67] reported that different particle sizes of corns in the mash or crumble feed did not affect flock uniformity. However, particle size is known to influence feed physicochemical properties, digestive efficiency, and gut development, which can lead to variability in nutrient utilization among birds [68,69]. Pelleting feeds can improve flock uniformity, but more studies are needed to investigate how different particle sizes of diverse ingredients may affect flock uniformity.

### 2.7. Heat Stress

Global warming and climate change pose significant challenges to broiler production by increasing environmental temperature and humidity within broiler houses [70]. Heat stress is well known to reduce growth performance and adversely affect the gut ecosystem in broiler chickens [71,72]. Several recent studies have clearly demonstrated that exposure to heat stress markedly decreased flock uniformity [73,74]. Variability in temperature and relative humidity within a broiler house (e.g., uneven air flow and temperature distribution) is considered one of the major factors contributing to reduced flock uniformity under heat stress conditions. In addition to elevated temperature and relative humidity, insufficient air flow (e.g., poor ventilation) represents a major factor contributing to heat stress in broiler chickens [75]. Poor ventilation and air flow can cause fluctuation in temperature and relative humidity within a broiler house, which can have varying effects on individual chickens. In addition, different coping capabilities of individuals against heat stress could lead to BW variation within a flock subjected to heat stress. A previous study by Gogoi et al. [76] showed that broilers with higher BW were more vulnerable to the heat stress compared to the broilers with lower and medium BW. Heat stress is well-known to reduce feed intake [77], leading to nutrient deficiencies that can differentially affect growth among individual birds, further contributing to BW variation within the flock. In addition, differences in the expression levels of heat shock factors and heat shock proteins, which respond to heat stress and mediate protective mechanisms, among individual birds could contribute to BW variations within a flock subjected to heat stress [78,79]. Moreover, elevated stress hormones (e.g., corticosterone) under heat stress can increase protein catabolism and alter energy metabolism, leading to greater variation in BW and reduced flock uniformity [75]. Hence, heat stress can negatively affect flock uniformity through a combination of environmental fluctuations, reduced feed intake, altered metabolic and hormonal responses, compromised gut health, and individual differences in physiological resilience.

### 2.8. Microbial Infection

**Figure 1:**
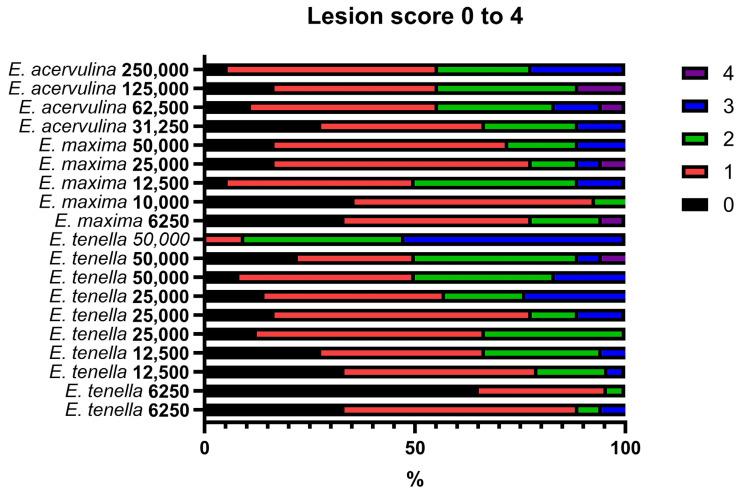
Variation in *Eimeria* lesion scores across different sections of the gastrointestinal tract (*E. acervulina*: duodenum; *E. maxima*: jejunum; *E. tenella*: ceca) was observed among broiler chickens administered identical doses of *Eimeria* spp. via oral gavage, as reported in various studies [87,88,89,90,91].

**Figure 2:**
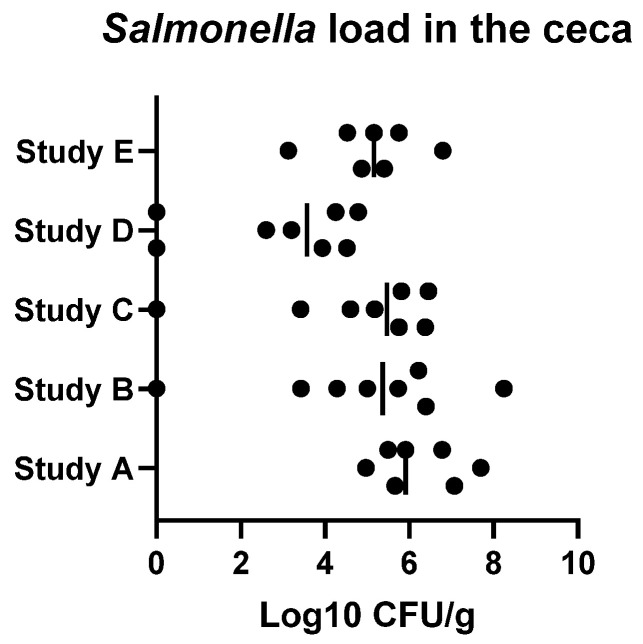
Variation in cecal *Salmonella* Typhimurium load on day 7 in broilers infected with 108 CFU/mL of S. Typhimurium on day 0, which was associated with differences in growth performance, as observed in our unpublished and published studies [86].

Broiler chickens are vulnerable to microbial infections due to unsanitary conditions in some commercial production system [80]. Various pathogens including *Salmonella* spp., *Campylobacter* spp., *Clostridium perfringens*, *Escherichia coli*, and *Enterococcus* spp., as well as protozoan parasites, can affect gut health, which results in the compromised growth performance. Among these, coccidiosis and necrotic enteritis (NE), caused by *Eimeria* spp. and/or *Clostridium perfringens*, are considered the primary enteric diseases that negatively impact growth rate, feed intake, and gut health, which may influence potentially flock uniformity [81,82,83]. Akram et al. [84] demonstrated that variations in intestinal morphology and the expression of genes associated with gut barrier function, nutrient transport, and oxidative processes may serve as preliminary factors influencing flock uniformity in broiler chickens. Reduced feed intake and compromised gut health, resulting from *Eimeria* spp. or *Clostridium perfringens* infections, can impair growth rate and ultimately contribute to decreased BW uniformity within a flock. Differences in birds’ resistance to infection constitute a key determinant of flock uniformity. In controlled experimental *Eimeria* inoculations via oral gavage, variations in lesion scores across different regions of the gastrointestinal tract are still observed among broilers, as demonstrated in our previous studies (Figure 1). Similarly, our NE infection model also exhibited variability among broilers when administered via oral gavage [82,85]. Furthermore, our previous unpublished and published studies [86] demonstrated that broiler chickens administered identical doses of *Salmonella* Typhimurium exhibited varying cecal Salmonella loads (Figure 2).

This summary indicates that individual broiler chickens exhibit varying resistance to microbial infections due to difference in individual immune system and gut physiology, and these differences contribute to increased BW variations. Boulton et al. [92] showed that phenotypic and genetic variation would induce response variation against *E. tenella* infection in broiler chickens. There are variations in the immune system in broiler population [93], and allelic variation and single nucleotide polymorphisms can determine susceptibility and resistance against microbial infection [92,94]. Based on these observations, it can be inferred that more severe or higher pathogen challenges could further exacerbate variations in flock uniformity. However, additional studies are needed to directly evaluate the relationship between pathogen load or challenge intensity and flock uniformity in commercial broiler populations.

As summarized in Table 2, multiple studies have reported that microbial infections adversely influence both growth performance and flock uniformity in broiler chickens. In controlled experimental settings where all individuals are oral-gavaged with identical doses of pathogens, responses can still vary among birds. However, under practical farm conditions, chickens are exposed to diverse microbial species and varying pathogen loads, which can further exacerbate reductions in flock uniformity. A previous study by Schwarz et al. [95] reported the presence of multiple *Eimeria* species and subspecies within a single farm. Notably, certain species and subspecies were identified as key contributors to pronounced reductions in broiler flock uniformity at the farm level. Collectively, these findings indicate that both heterogeneous pathogen exposure and individual variation in immune and gut physiology are key determinants of reduced flock uniformity in commercial broiler populations.

### 2.9. Vaccination Practices

In modern broiler production, birds are routinely vaccinated against various pathogens including virus, bacteria, and protozoa through mass vaccinations including spray, water, or feed administration [102,103,104]. Unlike individual administration, mass vaccination exposes the entire flock simultaneously, which can lead to variation in the effective dose received by each bird and consequently in the immunological response [105,106,107]. Additionally, intrinsic differences in the immune competence among individual birds contribute further to variability in vaccine efficacy [108]. Variations in vaccination administration, individual immune responses, and vaccination efficiency may negatively affect flock uniformity. Furthermore, variation in the development of protective immunity within a flock may increase susceptibility to disease outbreaks, thereby compromising overall flock performance and uniformity. To our knowledge, no studies have directly examined the impact of vaccine administration on flock uniformity, either in the presence or absence of pathogenic challenge. Additional research is warranted to address this gap.

## 3. Summary of Factors That Affect Flock Uniformity

**Figure 3:**
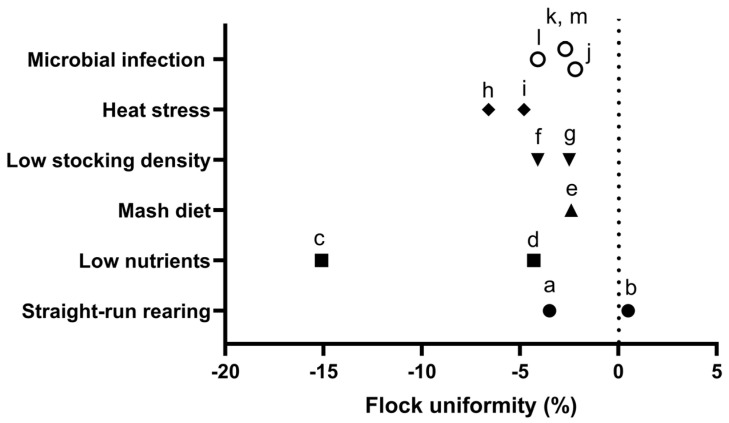
Decreased uniformity rate values (%) due to challenging conditions in the broiler production. Letters indicate the corresponding studies listed in Table 3.

Decreased uniformity rate values (%) with statistical significance (*p* < 0.05) due to challenging conditions in the broiler production are summarized in Figure 3 and in Table 3. Although the current literature base is insufficient to support a formal meta-analysis, existing studies collectively suggest consistent trends demonstrating that challenging conditions in poultry production negatively impact flock uniformity in broiler chickens. Values were expressed as uniformity rate, calculated using the formula $100\%-((\frac{SD\;of\;BW}{Mean\;of\;BW})$ × 100%). Flock uniformity ranged from 87.4% to 94.9%, while reductions under challenging conditions varied from −2.5% to −15.1%. Despite both studies examining similar conditions, discrepancies were observed in flock uniformity with one study reporting an increase [33] while the other showed a decrease under same-sex or straight-run rearing [34]. Most of the challenging conditions reduced approximately 5% uniformity rate compared to the control. In addition, Ahiwe et al. [63] demonstrated that the uniformity rate decreased by more than 5% when chickens were fed nutrient deficiency diets and exposed to heat stress, respectively. Collectively, these findings underscore that flock uniformity in broiler chickens is highly sensitive to multiple production challenges, emphasizing the importance of implementing effective management strategies to maintain uniformity.

## 4. Interrelationship Between the Factors Affecting Flock Uniformity

Various factors influence flock uniformity in broiler production, and these factors may interact with one another rather than acting independently. Several studies have reported no significant interactions among certain management and nutritional factors. A previous study by Xu et al. [67] observed no interaction between feed form and dietary coarse corn inclusion on flock uniformity. Similarly, Goo et al. [51] reported no interaction between stocking density and sex on flock uniformity. Nonetheless, England et al. [34] demonstrated an interaction between sex and reduced dietary crude protein level, indicating that the effects of nutritional strategies on flock uniformity may differ between males and females. Collectively, these findings suggest that interactions among factors affecting flock uniformity may depend on factors, genetics, production systems, environmental conditions, and experimental design. Therefore, further well-controlled and integrative studies are needed to elucidate complex interrelationships among nutritional, environmental, and management factors and to better predict their combined effects on flock uniformity.

## 5. Conclusions

Flock uniformity in broiler production is influenced by genetic, environmental, nutritional, and management factors. Under standard commercial conditions, uniformity typically reaches ~90% (D 14 to 49). Individual variation in growth, immune competence, and responses to environmental challenges can reduce the flock uniformity. Stressors such as heat, nutrient deficiencies, microbial infections, high stocking density, and mixed-sex rearing may further decrease uniformity by 2.5–15%, highlighting the sensitivity of flocks to multiple, interacting factors. Poor uniformity compromises production efficiency, feed utilization, animal welfare, and product quality. These observations underscore the need for research to understand the mechanisms underlying variability and to develop targeted strategies, including genetic selection, precision nutrition, and optimized management, to improve flock uniformity and overall productivity.

## Figures and Tables

**Table 1:** Effects of same-sex and straight-run rearing on the growth performance and flock uniformity in broiler production.

Strains	Observations Regarding Growth Performance and Uniformity	References
Ross 708	Straight-run rearing impaired growth performance and decreased flock uniformity compared to the same sex rearing. Same-sex rearing led to a tendency of increased flock uniformity from 0 to 49 days, whereas straight-run rearing exhibited a tendency to increase BW variation.	Da Costa et al. [31]
Cobb 500	While no differences were observed in the only male rearing and straight-run, but the straight-run rearing had higher final BW compared to the only female rearing.	Petkov et al. [32]
Ross	Straight-run rearing decreased final BW compared to the only male rearing and reduced flock uniformity compared to the only female rearing.	de Albuquerque et al. [33]
Cobb 500	Male and female had different crude protein requirements, and straight-run decreased uniformity in female chickens compared to the single sex rearing.	England et al. [34]

**Table 2:** Effects of microbial infection on the growth performance and flock uniformity of broiler chickens.

Challenging Conditions	Observations	References
5000 *E. acervulina* and 5000 *E. maxima* and 2500 *E. brunetti* on D 9.	Growth rate on D 35 ↓ (decreased) Flock uniformity on D 35 ↓	Sumon et al. [96]
5 birds were challenged with 100,000 *E. acervulina* and 60,000 *E. maxima*, and 7 birds were challenged with 25,000 *E. acervulina* and 5000 *E. maxima* on D 10.	Growth rate on D 35 ↓ Flock uniformity on D 35: no effects	Leung et al. [97]
5000 *E. acervulina* and 5000 *E. maxima* and 2500 *E. brunetti* on D 14. 1 mL of 4.5 × 10^7^ CFU/mL *C. perfringens* on D 15.	Growth rate on D 35 ↓ Flock uniformity on D 28 ↓	Ahiwe et al. [98]
5000 *E. acervulina* and 5000 *E. maxima* and 2500 *E. brunetti* on D 9. 1 mL of 10^8^ to 10^9^ CFU/mL *C. perfringens* on D 14.	Growth rate on D 35 ↓ Flock uniformity D 35: no effects	Xue et al. [99]
5000 *E. acervulina* and 5000 *E. maxima* and 2500 *E. brunetti* on D 15. 1 mL of 1 × 10^8^ CFU/mL *C. perfringens* on D 15.	Growth rate on D 35: no effects Flock uniformity on D 35 ↓	Kumar et al. [100]
Intraperitoneal injection of 3 mL of 100 µg/mL *Salmonella* Typhimurium lipopolysaccharides on D 13, 15, and 17.	Growth rate on D 35 ↓ Flock uniformity on D 28 ↓	Ahiwe et al. [101]

↓ indicates a significant decrease compared with the control.

**Table 3:** Summary of changes in flock uniformity (%) under different conditions in broiler chickens.

Symbol	Age	Control (%)	Experimental Group (%)	Change (%)	Conditions	References
a	D 45	92.1	88.6	−3.5	Same sex vs. straight run	de Albuquerque et al. [33]
b	D 34	90.3	90.8	0.5	Same sex vs. straight run	England et al. [34]
c	D 42	91.9	76.8	−15.1	Deficiency of energy and protein	Ahiwe et al. [63]
d	D 49	87.4	83.1	−4.3	Deficiency of amino acids	Corzo et al. [64]
e	D 14	91.0	88.6	−2.4	Mash vs. pelleted form	Xu et al. [67]
f	D 45	93.0	88.8	−4.1	Low vs. high stocking density	de Albuquerque et al. [33]
g	D 37	87.0	84.5	−2.5	Low vs. high stocking density	Feddes et al. [8]
h	D 42	89.4	85.8	−3.6	Heat stress	Archer [74]
i	D 42	93.1	88.3	−4.8	Heat stress	Ghasemi and Nari [73]
j	D 35	88.7	86.5	−2.2	Necrotic enteritis challenge	Ahiwe et al. [98]
k	D 35	91.5	88.8	−2.7	Eimeria challenge	Sumon et al. [96]
l	D 28	94.9	90.8	−4.1	Necrotic enteritis challenge	Kumar et al. [100]
m	D 28	94.5	91.8	−2.7	Salmonella lipopolysaccharide	Ahiwe et al. [101]

## Data Availability

No new datasets were generated or analyzed in this review.
